# Thyroid dysfunction and its association with micronutrients, lipids, and inflammation: an analytical case-control study

**DOI:** 10.1186/s12902-026-02275-1

**Published:** 2026-04-14

**Authors:** Dilshad Hamad Mustafa, Kochr Ali Mahmood, Faisal Faruq Sadiq, Saman Taher Barzinjy, Dawan Jamal Hawezy, Sirwan Khalid Ahmed

**Affiliations:** 1 College of Medicine, Hawler Medical University, Erbil, Kurdistan Region Iraq; 2 Faculty of General Medicine, Koya University, Koya, Kurdistan Region Iraq; 3 Medical Microbiology/Virology, Department of Medical Biochemical Analysis, Cihan University, Erbil, Kurdistan Region Iraq; 4 Department of Laboratory, Hawler Teaching Hospital, Ministry of Health, Erbil, Iraq; 5 College of Nursing, University of Raparin, Rania, Sulaymaniyah, Kurdistan Region 46012 Iraq

**Keywords:** Thyroid dysfunction, Micronutrients, Vitamin D, Zinc, Dyslipidaemia, C-reactive protein, Epstein–Barr virus

## Abstract

**Background:**

Biochemical, inflammatory, and immunological markers may provide insight into systemic alterations in individuals with or without thyroid dysfunction.

**Aim:**

This study compared a comprehensive biomarker profile between adults without thyroid disease and those with thyroid dysfunction, and examined gender differences, correlations, and predictive markers.

**Methods:**

In this case-control study, 120 adults without thyroid disease (cases) and 120 adults with thyroid dysfunction (controls) were enrolled. Demographics, anthropometry, and biochemical parameters—including thyroid hormones, micronutrients, lipid profiles, inflammatory markers, and Epstein–Barr virus PCR—were assessed. Between-group differences were analyzed using the Mann–Whitney U test and the χ² test. Spearman’s rho was used to determine correlations, and multivariable linear regression was used to identify predictors of group status.

**Results:**

Cases and controls were similar in age (60 ± 12 vs. 60 ± 10.5 years) and body mass index (BMI) (24.85 ± 3.69 vs. 25.58 ± 3.39 kg/m²). Compared with controls, cases had significantly lower zinc (97.98 vs. 143.03 µg/dL), vitamin D₃ (88.24 vs. 152.76 nmol/L), and HDL (88.75 vs. 152.25 mg/dL), but higher serum cholesterol (159.56 vs. 81.44 mg/dL), triglycerides (TG) (171.08 vs. 69.92 mg/dL), LDL (136.06 vs. 104.94 mg/dL), and CRP (140.05 vs. 100.95 mg/L) (all *p* < 0.001). In controls, TSH correlated positively with the Urea Breath Test (UBT) (rho = 0.334) and negatively with LDL (rho=–0.255). In cases, T3 showed a strong correlation with UBT (rho = 0.625) and a moderate correlation with PCR-EBV (rho = 0.417), while TSH correlated with UBT (rho = 0.434) and CRP (rho = 0.264). Regression analysis identified serum TG (β = 76.806, R²=0.462) and cholesterol (β = 64.834, R²=0.337) as the strongest predictors of case–control status.

**Conclusion:**

Adults without thyroid disease exhibit distinct biochemical and inflammatory profiles compared with those with thyroid dysfunction, highlighting lipid, micronutrient, and inflammatory markers as potential targets for risk stratification and clinical monitoring.

**Clinical trial number:**

Not applicable.

**Supplementary Information:**

The online version contains supplementary material available at 10.1186/s12902-026-02275-1.

## Introduction

Thyroid diseases are among the most prevalent endocrine disorders worldwide, exerting profound effects on metabolic, cardiovascular, and immune function [1]. These conditions encompass a broad spectrum, ranging from subclinical hypothyroidism and hyperthyroidism to overt thyroid dysfunction and autoimmune thyroid diseases (AITDs), such as Hashimoto’s thyroiditis and Graves’ disease [2]. The thyroid hormones triiodothyronine (T3) and thyroxine (T4) play a vital role in regulating energy metabolism, thermogenesis, and cellular activity, while thyroid-stimulating hormone (TSH), secreted by the anterior pituitary, governs their production [3]. Disturbances in T3 and T4 levels disrupt metabolic homeostasis, with far-reaching systemic consequences [4].

AITDs represent the leading cause of thyroid dysfunction, arising from an aberrant immune response against thyroid antigens and characterized by elevated anti-thyroid peroxidase and anti-thyroglobulin antibodies [5, 6]. Their pathogenesis involves a multifactorial interplay of genetic susceptibility, environmental triggers, and immune dysregulation [7]. Chronic low-grade inflammation has emerged as a crucial factor in the progression of thyroid disease, with elevated C-reactive protein (CRP) reflecting systemic inflammatory activity linked to both subclinical and overt dysfunction [8]. Oxidative stress, often secondary to inflammation, exacerbates thyroid tissue injury.

Micronutrients such as zinc and vitamin D play critical roles in maintaining thyroid integrity. Zinc is essential for thyroid hormone synthesis and function, whereas vitamin D exerts immunomodulatory effects that may attenuate autoimmune activity [9, 10].Vitamin D deficiency has been inversely associated with with the prevalence of thyroid autoantibodies suggesting a potential role in preserving immune tolerance [11]. Similarly, zinc deficiency can impair thyroid hormone metabolism, underscoring the biochemical interdependence between micronutrient status and thyroid function [12].

Dyslipidaemia is frequently observed in individuals with thyroid dysfunction, particularly hypothyroidism, which is associated with elevated low-density lipoprotein cholesterol (LDL-C) and triglycerides (TG), alongside reduced high-density lipoprotein cholesterol (HDL-C) [13]. This atherogenic profile elevates cardiovascular risk, driven by diminished lipoprotein lipase activity and altered hepatic lipid metabolism resulting from reduced thyroid hormone levels [14].

Infectious agents, particularly Epstein–Barr virus (EBV), have also been implicated in thyroid autoimmunity through mechanisms such as molecular mimicry and induction of autoreactive immune responses [15]. Polymerase chain reaction evidence demonstrating EBV DNA in thyroid tissues of patients with AITDs reinforces this potential link [16, 17].

Lifestyle factors further modulate thyroid health. Smoking has been consistently associated with increased incidence of both hyperthyroidism and hypothyroidism, mainly due to thiocyanate-induced inhibition of iodide uptake [18]. Obesity, often accompanied by metabolic syndrome, imposes additional strain on the hypothalamic–pituitary–thyroid axis, potentially exacerbating thyroid dysregulation [19, 20].

Despite extensive global research, the complex interplay between demographic variables, thyroid hormone profiles, micronutrient status, inflammatory markers, lipid abnormalities, and lifestyle factors has not been adequately characterized in Iraqi populations. Existing studies from Iraq are scarce, often limited by small sample sizes, lack of simultaneous assessment of multiple biochemical and immunological parameters, and minimal exploration of viral or micronutrient-related influences on thyroid health. This gap is significant given Iraq’s unique nutritional patterns, environmental exposures, and healthcare accessibility, which may distinctly shape thyroid disease presentation and progression.

The present study aims to address this evidence gap by examining the associations between thyroid dysfunction and micronutrient levels, lipid parameters, inflammatory status, and lifestyle factors.

## Methods

### Study design and setting

This was a hospital-based case–control study conducted in Erbil, Kurdistan Region–Iraq, between November 2023 and January 2024. Participants were recruited from tertiary hospitals and the Central Reference Laboratory. The cases consisted of adults with confirmed thyroid dysfunction, while the controls were adults with normal thyroid function and no history of thyroid disease. All participants underwent standardized biochemical, immunological, and molecular assessments.

### Participants

Participants were recruited using a consecutive, non-random sampling approach from tertiary care hospitals and the Central Reference Laboratory in Erbil, Kurdistan Region–Iraq [21]. Participants were classified into two groups: cases, defined as individuals with a confirmed diagnosis of thyroid dysfunction, and controls, defined as individuals without biochemical or clinical evidence of thyroid disease. Case identification was based on abnormal thyroid function tests (TSH, FT3, and/or FT4 outside reference ranges), positive anti-thyroid peroxidase (anti-TPO) and/or anti-thyroglobulin (anti-TG) antibodies, and corroborating endocrinology clinic documentation. Control eligibility required normal thyroid function tests, negative antibody results, and no history of thyroid or major endocrine disorders.

The inclusion criteria comprised adults aged 18 years or older who were willing to share their laboratory results for research purposes. Exclusion criteria included malignancy, chronic liver or kidney disease, pregnancy or lactation, use of thyroid-interfering medications (e.g., amiodarone, lithium) within the last six months, and acute infection at the time of sampling.

### Sample size determination

A priori power analysis was performed to ensure adequate statistical power for detecting clinically meaningful differences between cases and controls. Assuming a two-sided α = 0.05, 80% power, and a standardized mean difference (Cohen’s *d*) of 0.36 for continuous biomarkers (vitamin D, zinc, lipid profile), a minimum of 118 participants per group was required [21]. To account for attrition and to enable subgroup analyses, the final sample size was increased to 120 participants per group (*n* = 240). A secondary check confirmed that this sample size also provided sufficient power to detect group differences in categorical outcomes such as Epstein–Barr virus (EBV) seropositivity.

### Laboratory analyses

Blood samples were analyzed in accordance with standardized protocols and manufacturer guidelines. Thyroid hormones (T3, T4, and TSH) were measured using a fully automated chemiluminescent immunoassay (Cobas e411, Roche Diagnostics, Germany) with Elecsys commercial kits. Reference ranges were T3: 1.2–2.8 nmol/L, T4: 66–181 nmol/L, and TSH: 0.4–4.0 mIU/L. Thyroid autoantibodies (anti-TPO, anti-TG) were determined by ELISA (Bioassay Technology Laboratory, China), with cut-offs of > 35 IU/mL and > 40 IU/mL, respectively.

Micronutrients were assessed as follows: serum 25-hydroxyvitamin D [25(OH)D] by ELISA (DRG Instruments GmbH, Germany), categorized according to Endocrine Society guidelines (deficiency < 20 ng/mL, insufficiency 21–29 ng/mL, sufficiency ≥ 30 ng/mL), and serum zinc by atomic absorption spectrophotometry (AAnalyst 400, PerkinElmer, USA), with a reference range of 70–120 µg/dL.

Lipid profile parameters, including total cholesterol, triglycerides, high-density lipoprotein cholesterol (HDL-C), and calculated low-density lipoprotein cholesterol (LDL-C), were measured on an AU480 automated chemistry analyzer (Beckman Coulter, USA) using enzymatic colorimetric methods. Inflammatory status was evaluated via high-sensitivity C-reactive protein (hs-CRP) using particle-enhanced turbidimetric immunoassay (Cobas c501, Roche Diagnostics, Germany).

EBV status was determined by real-time PCR targeting the BamHI-W fragment (bioMérieux, France) following DNA extraction (Qiagen, Germany), expressed in viral copies/mL. Serological testing for EBV-specific antibodies (VCA-IgM, VCA-IgG, and EBNA-IgG) was performed using ELISA (Euroimmun, Germany) to differentiate between acute, past, and latent infections.

### Ethical considerations

The study protocol was approved by the Ethical Review Committee of the Medical Research Centre, Faculty of Medicine, Koya University (Reference No. 18/16, approved on 20 April 2025).

The requirement for informed consent was waived by the Ethics Committee because the study involved a retrospective analysis of routinely collected laboratory data, posed minimal risk to participants, and involved no identifiable personal information.

All data were fully anonymized prior to analysis, with personal identifiers removed and each participant assigned a unique study code. Data confidentiality was strictly maintained in accordance with institutional policies, national regulations, and the principles of the Declaration of Helsinki.

### Statistical analysis

Continuous variables were tested for normality using the Shapiro–Wilk test and are presented as mean ± standard deviation (SD) or median (interquartile range, IQR) as appropriate. Categorical variables are reported as frequencies and percentages. Between-group comparisons for continuous data employed the independent samples *t*-test or Mann–Whitney *U* test, while categorical data were compared using the χ² test. One-way analysis of variance (ANOVA) with post-hoc tests was used for comparisons across more than two groups. Correlations were assessed using Pearson’s or Spearman’s methods depending on the data distribution. All analyses were two-tailed, with *p* < 0.05 considered statistically significant, and were conducted using IBM SPSS Statistics, Version 27 (IBM Corp., Armonk, NY, USA).

## Results

### Participant characteristics

This study enrolled 240 participants: 120 cases with thyroid dysfunction and 120 controls without thyroid disease. The median age was 60 years in both groups (IQR 12 in cases vs. 10.5 in controls). Median height was 170 cm in both groups, while median weight was slightly lower among cases (70 kg [IQR 13.5]) compared with controls (72 kg [IQR 15]). Mean BMI was 24.85 kg/m² (SD 3.69) in cases and 25.58 kg/m² (SD 3.39) in controls. Males comprised 65.8% of cases and 62.5% of controls. Among cases, 6 (5.0%) participants were underweight, 51 (42.5%) had normal weight, 52 (43.3%) were overweight, and 11 (9.2%) had class I obesity. The corresponding figures in controls were 2 (1.7%), 56 (46.7%), 48 (40.0%), and 14 (11.7%) (Table 1).

**Table 1:** Characteristics of the participants (both groups)

Variables	Cases		Controls	
**Age**				
Median ± IQR	60 ± 12		60 ± 10.5	
**Height**				
Median ± IQR	170 ± 13		170 ± 10	
**Weight**				
Median ± IQR	70 ± 13.5		72 ± 15	
**BMI**				
Mean ± S.D	24.85 ± 3.69		25.58 ± 3.39	
Gender	Frequency	Percentage	Frequency	Percentage
Male	79	65.8	75	62.5
Female	41	34.2	45	37.5
BMI				
Under weight	6	5	2	1.7
Normal weight	51	42.5	56	46.7
Over weight	52	43.3	48	40
Obese 1	11	9.2	14	11.7

### Biochemical markers

Analysis of biochemical marker concentrations revealed significant differences between participants with thyroid dysfunction and the control group. Patients with thyroid dysfunction exhibited markedly reduced levels of zinc (97.98 ± 20.05 µg/dL vs. 143.03 ± 30.69 µg/dL, *p* < 0.001) and vitamin D₃ (88.24 ± 28.89 nmol/L vs. 152.76 ± 10.86 nmol/L, *p* < 0.001). Dyslipidaemia was prominent among cases, with higher mean total cholesterol (159.56 ± 58.3 mg/dL vs. 81.44 ± 38.35 mg/dL, *p* < 0.001), triglycerides (171.08 ± 56.0 mg/dL vs. 69.92 ± 23.83 mg/dL, *p* < 0.001), and LDL (136.06 ± 49.61 mg/dL vs. 104.94 ± 29.71 mg/dL, *p* < 0.001), alongside significantly lower HDL levels (88.75 ± 33.05 mg/dL vs. 152.25 ± 14.49 mg/dL, *p* < 0.001) compared with controls. Inflammatory markers also showed striking differences, as CRP was substantially elevated in cases (7.29 ± 14.17 mg/L) compared to controls (2.09 ± 0.82 mg/L, *p* < 0.001). Although PCR-EBV values were higher among cases (127.5 ± 26.5 copies/mL) than controls (113.5 ± 13.25 copies/mL), this difference was not statistically significant (*p* = 0.125). Collectively, these findings indicate that thyroid dysfunction is strongly linked to micronutrient deficiencies, lipid abnormalities, and increased systemic inflammation (Table 2).

**Table 2:** Biochemical markers concentrations in participants with and without thyroid dysfunction

Biochemical markers	Cases (*n* = 120) Mean ± SD	Controls (*n* = 120) Mean ± SD	*P* value
Zinc (µg/dL)	97.98 ± 20.05	143.03 ± 30.69	< 0.001
Vitamin D₃ (nmol/L)	88.24 ± 28.89	152.76 ± 10.86	< 0.001
Serum cholesterol (mg/dL)	159.56 ± 58.3	81.44 ± 38.35	< 0.001
Triglyceride (mg/dL)	171.08 ± 56	69.92 ± 23.83	< 0.001
LDL (mg/dL)	136.06 ± 49.61	104.94 ± 29.71	< 0.001
HDL (mg/dL)	88.75 ± 33.05	152.25 ± 14.49	< 0.001
PCR-EBV (copies/mL)	127.5 ± 26.5	113.5 ± 13.25	0.125
CRP (mg/L)	7.29 ± 14.17	2.09 ± 0.82	< 0.001

### Gender-specific biochemical markers profiles

Table 3 presents the gender-specific biomarker concentrations among control and case groups. In the control group, males had significantly higher mean T4 levels (92.24 ± 33.08 vs. 77.60 ± 16.32 nmol/L, *p* = 0.007), zinc concentrations (104.09 ± 26.91 vs. 89.53 ± 34.58 µg/dL, *p* = 0.011), and vitamin D₃ levels (41.31 ± 12.41 vs. 35.69 ± 6.34 nmol/L, *p* = 0.006) compared to females. No other biomarkers showed significant gender-related differences in the control group. In the case group, none of the biomarkers demonstrated statistically significant differences between males and females, although trends were noted for T3 (*p* = 0.082) and triglycerides (*p* = 0.092).

**Table 3:** Gender-specific biomarker concentrations in control and case groups

Biomarkers	Control – Female (*n* = 45) Mean ± SD	Control – Male (*n* = 75) Mean ± SD	*p*-value	Cases – Female (*n* = 41) Mean ± SD	Cases – Male (*n* = 79) Mean ± SD	*p*-value
T3 (nmol/L)	2.46 ± 1.12	3.01 ± 5.23	0.490	2.58 ± 1.15	3.03 ± 1.40	0.082
T4 (nmol/L)	77.60 ± 16.32	92.24 ± 33.08	0.007	119.29 ± 48.72	107.37 ± 44.30	0.179
TSH (mIU/L)	2.41 ± 0.99	2.20 ± 1.19	0.337	2.26 ± 1.64	2.77 ± 1.57	0.101
Anti-TPO (IU/mL)	24.09 ± 6.90	22.16 ± 8.57	0.203	26.93 ± 32.36	29.08 ± 12.26	0.601
Anti-TG (IU/mL)	34.33 ± 18.11	31.99 ± 15.03	0.445	104.51 ± 324.41	59.81 ± 42.29	0.229
Zinc (µg/dL)	89.53 ± 34.58	104.09 ± 26.91	0.011	79.85 ± 23.15	80.19 ± 18.40	0.931
Vitamin D₃ (nmol/L)	35.69 ± 6.34	41.31 ± 12.41	0.006	28.44 ± 11.71	29.13 ± 12.29	0.768
Serum cholesterol (mg/dL)	119.00 ± 42.12	128.39 ± 35.74	0.196	197.93 ± 72.05	185.53 ± 49.72	0.271
Triglycerides (mg/dL)	90.87 ± 17.74	94.47 ± 26.87	0.426	181.00 ± 68.61	162.84 ± 47.51	0.092
LDL (mg/dL)	81.64 ± 25.21	84.08 ± 32.25	0.666	113.73 ± 50.72	102.01 ± 48.88	0.221
HDL (mg/dL)	46.27 ± 13.21	47.23 ± 15.29	0.727	30.95 ± 16.71	34.15 ± 17.67	0.340
PCR-EBV (copies/mL)	0.00 ± 0.00	0.00 ± 0.00	–	4.54 ± 18.54	37.94 ± 206.08	0.303
CRP (mg/L)	1.97 ± 0.77	2.17 ± 0.85	0.198	6.35 ± 10.76	7.78 ± 15.70	0.603

### Correlation analysis

In the control group, T3 demonstrated a weak positive correlation with the Urea breath test (rho = 0.215; *p* = 0.018). T4 showed a weak positive correlation with CRP (rho = 0.223; *p* = 0.014). TSH had a moderate positive correlation with the Urea breath test (rho = 0.334; *p* < 0.001), a weak positive correlation with vitamin D3 (rho = 0.210; *p* = 0.021), and a weak negative correlation with S.TG (ρ = −0.255; *p* = 0.005) (Table 4).

**Table 4:** Spearman’s rank correlation coefficient between thyroid and biochemical markers in the control group

Variables			Urea breath test	Anti-TPO	Anti- TG	ZINC	Vit D3	S.CHO	S.TG	LDL	HDL	CRP
Spearman’s rho	T3	rho	0.215	-0.006	0.088	0.098	0.021	0.081	0.052	-0.155	-0.131	0.121
		P value	0.018	0.945	0.339	0.286	0.816	0.380	0.572	0.090	0.153	0.188
	T4	rho	-0.015	-0.044	-0.025	0.140	-0.003	-0.005	0.033	0.052	-0.157	0.223
		P value	0.868	0.634	0.789	0.127	0.971	0.958	0.717	0.570	0.087	0.014
	TSH	rho	0.334	-0.206	0.092	-0.105	0.210	0.036	-0.255	0.073	-0.009	0.004
		P value	< 0.001	0.024	0.316	0.255	0.021	0.694	0.005	0.426	0.924	0.968

S.CHO, serum cholesterol; S.TG, serum triglycerides; LDL, low-density lipoprotein; HDL, high-density lipoprotein; CRP, C-reactive protein; PCR-EBV, Epstein–Barr virus DNA polymerase chain reaction; Anti-TPO, anti-thyroid peroxidase antibody; Anti-TG, anti-thyroglobulin antibody; Vit D₃, vitamin D₃; rho, Spearman’s rank correlation coefficient

In the case group, T3 showed a strong positive correlation with the Urea breath test (rho = 0.625; *p* < 0.001), a weak positive correlation with anti-TPO (rho = 0.194; *p* = 0.034), a weak negative correlation with zinc (rho = − 0.191; *p* = 0.037), a moderate positive correlation with PCR-EBV (rho = 0.417; *p* < 0.001), and a weak positive correlation with CRP (rho = 0.209; *p* = 0.022). T4 had a weak positive correlation with vitamin D3 (rho = 0.254; *p* = 0.005) and with PCR-EBV (rho = 0.202; *p* = 0.027). TSH demonstrated a moderate positive correlation with the Urea breath test (rho = 0.434; *p* < 0.001), a weak positive correlation with vitamin D3 (rho = 0.224; *p* = 0.014), a moderate positive correlation with PCR-EBV (rho = 0.293; *p* = 0.001), and a weak positive correlation with CRP (rho = 0.264; *p* = 0.004) (Table 5).

**Table 5:** Spearman’s rank correlation coefficients between thyroid and biochemical markers in the control group

Variables			Urea breath test	Anti-TPO	Anti- TG	ZINC	Vit D3	S.CHO	S.TG	LDL	HDL	PCR-EBV	CRP
Spearman’s rho	T3	rho	0.625	0.194	0.153	-0.191	-0.036	0.062	-0.025	-0.043	0.177	0.417	0.209
		P value	< 0.001	0.034	0.096	0.037	0.698	0.499	0.786	0.643	0.053	< 0.001	0.022
	T4	rho	0.168	0.030	-0.095	0.069	0.254	0.086	0.073	-0.060	-0.178	0.202	0.063
		P value	0.067	0.744	0.302	0.457	0.005	0.352	0.427	0.513	0.051	0.027	0.492
	TSH	rho	0.434	0.134	0.077	-0.052	0.224	0.080	0.055	-0.101	0.145	0.293	0.264
		P value	< 0.001	0.144	0.403	0.572	0.014	0.386	0.554	0.270	0.114	0.001	0.004

S.CHO, serum cholesterol; S.TG, serum triglycerides; LDL, low-density lipoprotein; HDL, high-density lipoprotein; CRP, C-reactive protein; PCR-EBV, Epstein–Barr virus DNA polymerase chain reaction; Anti-TPO, anti-thyroid peroxidase antibody; Anti-TG, anti-thyroglobulin antibody; Vit D₃, vitamin D₃; rho, Spearman’s rank correlation coefficient

### Regression analysis

Multivariable linear regression analysis (Table 6) revealed that, after adjusting for covariates, cases had significantly lower zinc (β = − 18.246; *p* < 0.001; R² = 0.160), vitamin D₃ (β = − 10.465; *p* < 0.001; R² = 0.193), and HDL (β = − 14.030; *p* < 0.001; R² = 0.165) levels compared with controls. Conversely, cases had higher serum cholesterol (β = 64.834; *p* < 0.001; R² = 0.337), serum TG (β = 76.806; *p* < 0.001; R² = 0.462), LDL (β = 22.298; *p* < 0.001; R² = 0.103), and CRP (β = 4.312; *p* < 0.001; R² = 0.285) (Table 6).

**Table 6:** Multivariable linear regression analysis of biochemical markers in cases and controls

Biochemical markers	β (Case vs. Control)	*p*-value	*R*²
Zinc	–18.246	< 0.001	0.160
Vitamin D₃	–10.465	< 0.001	0.193
Serum cholesterol	64.834	< 0.001	0.337
Serum triglycerides	76.806	< 0.001	0.462
LDL	22.298	< 0.001	0.103
HDL	–14.030	< 0.001	0.165
PCR-EBV	22.290	0.125	0.161
CRP	4.312	< 0.001	0.285

## Discussion

This study reveals important associations between demographic features and hormone, biochemical, and inflammatory markers in thyroid conditions which indicate their complex relationships. The study successfully achieved its primary objective by identifying significant differences between patients and healthy participants in micronutrient levels, as well as lipid test results and inflammatory indicators.

The results demonstrated that zinc and vitamin D3 levels were significantly lower in case participants compared to control participants. The current study confirms previous research which suggests that micronutrients play a role in regulating thyroid function [12, 22, 23]. Thyroid hormone production and function decrease when zinc deficiency exists as it intensifies the development of thyroid disease, and vitamin D3 deficiency creates pathways for the etiopathogenesis of AITDs [24]. The extensive reduction of these micronutrients in patients suggests that Zn and vitamin D3 have beneficial effects on thyroid function; however, their deficiency may create conditions that make patients susceptible to disease [25].

The case group exhibited marked dyslipidemia through elevated levels of total cholesterol and triglycerides along with increased LDL and reduced HDL. Research confirms previous findings that link hypothyroidism to lipid metabolism disorders [3, 26]. Thyroid hormones regulate essential enzymes responsible for lipid metabolism; therefore, hypothyroidism leads to harmful lipid profiles, which increase the risk of cardiovascular disease [27]. The mechanistic relationship between lipid alterations and thyroid disease becomes more evident through TSH, T4 and serum cholesterol interactions.

Systemic inflammation served as an essential element among the case patients. PCR-EBV and CRP values from the case group showed significant elevation indicating both chronic inflammation and possible viral reactivation. Research findings support the theory that autoimmune thyroid disorders arise from chronic inflammation often in conjunction with Epstein-Barr Virus (EBV) infections [28, 29]. The elevated associations between inflammatory biomarkers and thyroid function tests (T3, TSH) demonstrate that immune reactions along with viral agents contribute to the development of thyroid dysfunction.

Intriguingly, in our case group, men exhibited higher anti-TPO levels than women—a finding that diverges from established epidemiological patterns. Most prior studies report both a higher prevalence and titres of anti-TPO in women. For example, women have nearly 2.5-fold greater odds of TPO-Ab positivity, and tend to demonstrate significantly higher mean titres [30]. The discrepancy in current data may stem from sample characteristics of participants such as size, geographic or genetic factors, or potential differences in treatment history between men and women. Additionally, the cross-sectional design limits causal inference and may capture a transient antibody fluctuation rather than actual long-term trends. Further investigation especially in larger, longitudinal cohorts will be necessary to determine whether this pattern is unique to our population or represents new epidemiological trend.

The control female participants demonstrated lower T4 levels along with lower Zn and vitamin D3 concentrations compared to male participants according to a previous study, indicating that women experience higher rates of micronutrient deficiencies and thyroid problems [31]. The study findings demonstrate that gender must be considered during both risk evaluation and treatment planning for thyroid disease.

The correlation study demonstrated how different factors including infection and inflammation interact with thyroid metabolism in complex ways. The case group patients showed a robust positive relationship between T3 levels and both Urea Breath Test and PCR-EBV results indicating that Helicobacter pylori infection and viral reactivation could affect thyroid hormone production. Systemic inflammation was found to affect thyroid regulation because TSH showed positive associations with both CRP and PCR-EBV. Previous research has demonstrated that Helicobacter pylori infections and viral infections lead to autoimmune thyroiditis [32].

The weak negative relationship between T3 and vitamin D3 levels suggests that vitamin D3 deficiency indirectly affects thyroid function through its immunomodulatory action, rather than its role in thyroid hormone synthesis [33, 34].

This study concludes by demonstrating that thyroid dysfunction results from multiple causes, including nutritional deficiencies, lipid changes, chronic inflammation, viral infections, and sex differences. The cross-sectional nature of the study prevents the conclusion of cause-and-effect relationships, but it allows researchers to establish the strength of association for identifying both intervention and longitudinal study targets.

The study encompassed multiple areas of strength and conducted a detailed analysis of demographic factors, along with hormone levels, micronutrient levels, inflammation markers, and thyroid status, to create a comprehensive picture of thyroid determinants. The research achieved sufficient statistical power through its available cases and controls, combined with an adequate sample size for comparison. Standardized laboratory assays, combined with strict data collection protocols, ensured both the reliability and validity of the biochemical information. The extensive correlation analysis provided a deeper understanding of how micronutrient status interacts with infection, inflammation, and thyroid hormone regulation.

There are some limitations, including the study design, which is a cross-sectional study, preventing researchers from establishing cause-and-effect relationships between observed variables, so it remains unclear whether biochemical changes lead to thyroid dysfunction. The research data originated from a single geographic area, which limits the applicability of the results to different populations with varying genetic profiles, nutritional patterns, and environmental conditions. The study failed to properly examine the effects of dietary intake and sun exposure on vitamin D3 status, as well as the type or severity of thyroid illness, as potential confounding variables. The study excluded certain medical conditions and medications, which might have resulted in selection bias. The study needs future longitudinal studies to confirm and verify its findings.

## Conclusion

The current study suggests that thyroid dysfunction is strongly linked to Zn and vitamin D3 deficiency, dyslipidemia, elevated inflammatory markers, and an increased risk of viral infections. The results suggest that thyroid disease has multiple causes, including nutritional, metabolic, immune system, and infectious factors.

Gender differences were also noted, which indicates the importance of gender-specific health interventions. These findings provide significant input to regional knowledge of thyroid diseases and highlight the need for an interdisciplinary approach to diagnosis, treatment, and prevention focusing on modifiable risk factors.

Our findings indicate that elevated EBV markers are associated with thyroid dysfunction and heightened inflammatory status. However, due to the cross-sectional study design, these results should be interpreted as observational associations only, and causality cannot be inferred. Prospective cohort or mechanistic studies are warranted to clarify the direction and nature of this relationship.

The research suggests that micronutrient deficiency screening should be included in the assessment of thyroid disorders, alongside regular screening of lipid profiles and markers of inflammation. Special emphasis should be given to women because they might be at greater risk for deficiency as well as AITDs. Public health strategies to promote good nutrition, specifically through vitamin D and Zn supplementation, should be sought out. Furthermore, screening for conditions such as Helicobacter pylori and Epstein-Barr virus infection in patients with thyroid dysfunction may provide potential avenues for early treatment. Future studies should utilize prospective cohort study designs to increase causality and determine the potential for targeted supplementation and anti-inflammatory therapies to improve thyroid outcomes.

## Relevant to Sustainable Development Goals (SDGs)

This study aligns with the UN Sustainable Development Goals, particularly SDG 3 (Good Health and Well-Being) through its focus on non-communicable disease prevention, SDG 2 (Zero Hunger) by addressing micronutrient deficiencies, and SDG 5 (Gender Equality) by highlighting gender-based health differences. It also supports SDG 9 (Innovation), SDG 10 (Reduced Inequalities), and SDG 17 (Partnerships) through scientific collaboration and regional health advancement.

## Supplementary Information

Below is the link to the electronic supplementary material.

Supplementary Material 1

## Data Availability

The data is available upon request to the corresponding author.
